# The Impact of Cyber-Ostracism on Bystanders’ Helping Behavior Among Undergraduates: The Moderating Role of Rejection Sensitivity

**DOI:** 10.3390/bs15081120

**Published:** 2025-08-18

**Authors:** Qian Sun, Shuchang Su, Shuting Lai, Xi Ding, Shaoyang Guo

**Affiliations:** 1 Department of Psychology, Suzhou University of Science and Technology, Suzhou 215009, China; 2 School of Educational Science, Yangzhou University, Yangzhou 225012, China

**Keywords:** cyber-ostracism, bystanders’ helping behavior, rejection sensitivity

## Abstract

This study examined how undergraduate bystanders respond to cyber-ostracism events and the moderating role of rejection sensitivity in shaping helping behaviors using two experiments. In Experiment 1 (*N* = 276), we first measured participants’ rejection sensitivity, then manipulated cyber-ostracism using a social media interaction scenario, and finally, measured helping behavior towards the (non-)ostracism target using a questionnaire. Experiment 2 (*N* = 258) sought to replicate and extend the findings of Experiment 1 using a methodologically refined design, in which we employed a modified Cyberball paradigm to manipulate cyber-ostracism and measured bystanders’ helping behavior through their resource allocation decisions (i.e., token sharing). The results revealed that witnessing cyber-ostracism significantly promoted bystanders’ helping behavior. This facilitative effect was more pronounced among bystanders with high rejection sensitivity. These findings shed light on the psychological mechanisms underlying bystanders’ helping responses in the context of cyber-ostracism and provide a new perspective for understanding interpersonal interactions in digital environments.

## 1. Introduction

### 1.1. Cyber-Ostracism and Its Impact

Ostracism is an inevitable and universal phenomenon in social interactions. It refers to a socio-psychological phenomenon wherein individuals are rejected, isolated, or ignored by others or social groups, posing a threat to an essential need for belonging and interpersonal connection (Williams, 2007, 2009). With the development of internet technology, the online world has become a significant arena for social interaction. While opportunities for interaction increase, so does the likelihood of being ignored or excluded from certain circles, giving rise to cyber-ostracism. 

Cyber-ostracism refers to the negative experience arising from a lack of expected feedback in online interactions (Lutz & Schneider, 2021; Schneider et al., 2017; X. Sun et al., 2017). This encompasses a range of specific online behaviors that signal exclusion to the target, such as messages being read but not replied to (Lutz, 2022), posts receiving no comments or likes (Lutz & Schneider, 2021), being ignored in group chats (Donate et al., 2017), or being removed from or blocked within social circles (Hatun & Demirci, 2022; Qin et al., 2025). While the salience and interpretation of these signals may vary across platforms—for instance, read receipts make non-reply more explicit than on platforms lacking this feature—the core experience of perceived interpersonal rejection triggered by feedback absence constitutes cyber-ostracism.

Existing research has focused almost exclusively on how cyber-ostracism harms its direct victims (i.e., the ostracized). According to Williams’ (2009) Need–Threat Model, ostracism triggers immediate threats to fundamental needs—belonging, self-esteem, control, and meaningful existence—motivating coping responses. Consistent with this framework, empirical studies demonstrate that cyber-ostracism is associated with increased depression (H. Ding et al., 2022; Lei et al., 2018; Niu et al., 2018), social anxiety (Galbava et al., 2021; L. Shi et al., 2025), aggressive behavior (Jin et al., 2024; Xing & Kuo, 2024), and online deviance (C. Shi et al., 2023).

### 1.2. The Role of Bystanders in Cyber-Ostracism

However, this victim-centric lens neglects a critical force shaping cyber-ostracism events: the bystander, i.e., third-party observers witnessing but not directly involved in the exclusion. As prevalent participants in digital spaces (Huang et al., 2019), bystanders’ actions, such as helping the ostracized or reinforcing ostracism, significantly affect the psychological impact of those targeted. Helping behavior, defined as voluntary actions intended to assist others and alleviate their distress (Stukas & Clary, 2012), represents a key prosocial response bystanders might engage in. Whether and which bystanders translate observation into such help when confronted with cyber-ostracism remains unknown. Investigating bystanders’ behavioral responses in the population offers significant potential for social intervention by identifying avenues to mitigate the harm of cyber-ostracism.

Moreover, although cyber-ostracism occurs across the lifespan, it carries heightened significance for undergraduate populations. As the demographic most engaged in internet-based communication (CNNIC, 2023; Xing & Kuo, 2024)—and critically, as emerging adults navigating Erikson’s (1963) intimacy vs. isolation developmental stage—undergraduates exhibit amplified vulnerability to cyber-ostracism. Considering this, the present study focuses specifically on undergraduate bystanders, examining their subsequent helping behaviors toward the ostracized and the factors influencing these responses after witnessing cyber-ostracism.

### 1.3. Theoretical Framework and Hypotheses

#### 1.3.1. Cyber-Ostracism and Bystanders’ Helping Behavior

Research into offline contexts demonstrates that witnessing social exclusion threatens individuals’ basic psychological needs (e.g., self-esteem, sense of control), triggering negative psychological experiences (Poon et al., 2020; Y. Sun et al., 2023; Yang & Zou, 2020; Zhu et al., 2025). To restore their psychological state, bystanders typically employ two strategies: (1) supporting the excluded through empathic attention or resource provision (Masten et al., 2011; Paolini et al., 2017; Wesselmann et al., 2013; Zou et al., 2022) or (2) punishing the excluder through moral derogation or monetary sanctions (F. Guo et al., 2022; Rudert et al., 2018, 2020). Although cyber-ostracism lacks physical presence and nonverbal cues, it shares core mechanisms for triggering interpersonal threat with face-to-face exclusion (Büttner & Lutz, 2025; Qin et al., 2025; Williams, 2007). Consequently, after witnessing cyber-ostracism, individuals may be more likely to exhibit compensatory or helping behaviors towards cyber-ostracized individuals. Consistent with this speculation, Bernstein et al. (2018) found that bystanders in a simulated online classroom showed stronger learning and befriending intentions towards cyber-ostracized individuals rather than the included individuals. Considering the evidence above, we proposed the following hypothesis:

**H1:**  *Bystanders will exhibit more helping behaviors towards targets of cyber-ostracism than towards non-ostracized individuals.*

#### 1.3.2. Rejection Sensitivity as a Moderator

Furthermore, bystanders’ responses to cyber-ostracism are not uniform but vary based on individual traits. Rejection sensitivity, a trait directly pertinent to interpersonal experiences, denotes an individual’s heightened vigilance and anxious expectation of rejection cues (X. Ding et al., 2020; Downey & Feldman, 1996; Zhang et al., 2021). Social-cognitive models of rejection sensitivity (Downey & Feldman, 1996; Levy et al., 2001) suggest that individuals with low rejection sensitivity are less responsive to interpersonal rejection cues and less influenced by them; conversely, individuals with high rejection sensitivity are prone to perceiving ambiguous social cues as rejection due to their heightened sensitivity to perceived interpersonal threats. This sensitivity can trigger significant negative emotional and behavioral consequences, including exacerbated loneliness (Lyu et al., 2024), increased depression (Zhou et al., 2020), heightened risk of self-injurious behaviors and suicidality (Berenson et al., 2009), or aggression towards the source of rejection (Bondü & Richter, 2016).

Social projection theory (Krueger, 2008) posits that individuals tend to project their own attitudes, emotions, and traits onto others, creating a tendency to “judge others by oneself.” Consequently, we hypothesize that when witnessing cyber-ostracism, bystanders with high (vs. low) rejection sensitivity are more likely to project their own painful rejection experiences and strong reactions onto the excluded target. This projection process is expected to amplify bystanders’ perception of the threat experienced by the target and enhance empathic understanding of their thwarted needs, potentially prompting increased helping behaviors towards the excluded individual. Therefore, we hypothesized the following:

**H2:**  *Rejection sensitivity positively moderates the relationship between cyber-ostracism and bystanders’ helping behavior. Specifically, bystanders with high rejection sensitivity will exhibit a more significant increase in helping behavior after witnessing cyber-ostracism compared with those with low rejection sensitivity.*

### 1.4. Overview of the Present Research

We performed two experiments to evaluate these hypotheses. In Experiment 1, we measured participants’ rejection sensitivity first, then exposed them to randomized text-based social media scenarios depicting ostracism/non-ostracism, and assessed helping behavior via a questionnaire. Experiment 2 advanced the methodological design to cross-validate and extend the findings of Experiment 1. Specifically, we used a modified Cyberball paradigm to manipulate cyber-ostracism for higher ecological validity, and measured actual helping behavior through resource allocation (token sharing) to mitigate social desirability bias.

## 2. Experiment 1

### 2.1. Participants and Design

An a priori power analysis using G*Power 3.1 (Faul et al., 2007) determined that a minimum sample size of 210 participants was necessary to achieve 95% power (1 − β = 0.95) for detecting a medium effect size (f = 0.25) with α = 0.05. To exceed this minimum and account for potential exclusions, we recruited participants over a 4-week period from 1 March to 1 April 2024. Ultimately, we recruited 276 undergraduate students (189 females) aged 18–25 years (*M* = 19.72, *SD* = 1.46) from Suzhou University of Science and Technology via the campus psychology participant pool and the campus online recruitment platform. Using random assignment, 138 participants were allocated to the ostracism condition (*N*_female_ = 93; *M*_age_ = 19.74, *SD* = 1.39) and 138 to the control condition (i.e., non-ostracism; *N*_female_ = 96; *M*_age_ = 19.70, *SD* = 1.53).

All participants were required to be full-time undergraduates and meet the following inclusion criteria: no psychiatric/neurological history, non-psychology majors, and no prior psychology coursework or participation in similar experiments. All provided written informed consent after reading a detailed information sheet regarding the research purpose, procedures, risks, withdrawal rights without penalty, etc. Participants were explicitly assured that their data would be anonymized (via numeric codes), kept strictly confidential, and used solely for research purposes. This experiment complied with the principles of the 1964 Helsinki Declaration, including subsequent amendments, and received approval from the Human Research Ethics Committee of Suzhou University of Science and Technology.

### 2.2. Materials

#### 2.2.1. Manipulation of Cyber-Ostracism

Drawing on previous research (Bernstein et al., 2018; Y. Guo et al., 2020), cyber-ostracism was manipulated using an event imagination paradigm. Participants were instructed to read and vividly imagine a social media interaction scenario; specifically, acting as observers, participants received one of two text-based scenario materials. The material for the ostracism condition was “Today, Person A shared a vlog about visiting an ancient town in Suzhou on WeChat Moments. Person B liked the post and commented. Shortly after, Person C also liked and commented. Person A quickly responded to Person B. However, after waiting a full day, Person C received no reply from Person A.” The material for the non-ostracism condition was “Today, Person A shared a vlog about visiting an ancient town in Suzhou on WeChat Moments. Person B liked the post and commented. Shortly after, Person C also liked and commented. Person A quickly responded to both Person B and Person C.”

#### 2.2.2. Manipulation Checks of Cyber-Ostracism

In line with Fiset et al. (2017), participants completed three items evaluating cyber-ostracism after reading the scenario, specifically assessing feelings that Person C was disliked, isolated, or unable to fit into the group. Responses were measured using a 5-point Likert scale ranging from 1 (strongly disagree) to 5 (strongly agree). A composite manipulation check score was derived by averaging responses from the three items, where higher scores reflected increased perceived severity of cyber-ostracism.

#### 2.2.3. Measurement of Rejection Sensitivity

Participants’ rejection sensitivity was measured using the Chinese version of the Rejection Sensitivity Questionnaire (Li, 2007). The 17-item tool utilizes a 5-point Likert scale ranging from 1 (very unlikely) to 5 (very likely), with six items (1, 2, 4, 9, 10, 17) reverse-scored to mitigate response bias. Example items include “I am overly sensitive to rejection” and the reverse-coded “I do not care much whether others accept or reject me.” The rejection sensitivity index was determined by the total score, with higher scores reflecting increased sensitivity to rejection. The scale in Experiment 1 showed acceptable internal consistency with a Cronbach’s α of 0.761.

#### 2.2.4. Measurement of Bystanders’ Helping Behavior

The measurement was operationalized as participants’ self-reported behavioral propensity to assist the target individual (Person C) in future interactions. This construct was measured using a three-item scale adapted from the Bystander Intentions to Help the Victim Scale (Bastiaensens et al., 2014). The items included the following: (1) “I will tell Person C that I believe cyber-ostracism behavior is not OK,” (2) “I will make every effort to console Person C,” and (3) “I will provide Person C with constructive advice.” Responses were recorded on a 5-point Likert scale, with the sum score serving as the helping behavior index. Higher scores indicate greater willingness to engage in bystander intervention. The scale exhibited strong internal consistency in this study, with a Cronbach’s alpha of 0.849.

### 2.3. Procedures

Participants volunteered and understood that the study aimed to examine bystanders’ reactions to observed online interactions. Upon arrival at the laboratory, participants completed experimental tasks via laboratory computers, which presented instructions and recorded behavioral responses. After reading the instructions, participants sequentially completed (1) measurement of rejection sensitivity, (2) manipulation of cyber-ostracism and its manipulation checks, and (3) measurement of helping behavior. To minimize demand characteristics, they were explicitly asked to answer honestly and attentively and were assured that their responses were anonymous and could not be traced back to them.

Subsequently, participants provided demographic information (gender, age, daily internet usage) and received a comprehensive debriefing. A trained experimenter verbally explained and displayed on-screen that all online interactions (including those involving Persons A, B, and C) involved computer-simulated avatars unrelated to real social relationships. The clear research purpose was then disclosed, and participants were invited to ask questions. To mitigate potential discomfort, we provided two free, confidential support options: (1) email access to a China-certified Level-3 Psychological Consultant (free service, typical response within 8 h), and (2) Jiangsu Province College Students 24-h Psychological Hotline (toll-free). No participant requested additional support. 

Finally, participants were thanked and received 5 CNY (≈0.70 USD) as compensation. The entire procedure lasted approximately 10 min. 

### 2.4. Statistical Analysis

We conducted analyses using IBM SPSS 29.0 and the PROCESS macro v4.1 (Hayes, 2022). After verifying normality violations using the Shapiro–Wilk tests, we assessed variable relationships using Pearson’s correlations (normally distributed variables) or Spearman’s correlations (non-normally distributed variables) and performed group comparisons with independent-samples *t*-tests (normally distributed variables) or Mann–Whitney *U*-tests (non-normally distributed variables). For moderation analysis, we tested whether rejection sensitivity influences the link between cyber-ostracism (coded as non-ostracism = 0, ostracism = 1) and bystanders’ helping behavior using the PROCESS macro (Model 1; Hayes, 2022). Rejection sensitivity, a continuous variable, was mean-centered to mitigate multicollinearity. Statistical significance was determined using 5000 bootstrap samples generating 95% bias-corrected confidence intervals (CIs), with effects considered significant when intervals excluded zero (Preacher & Hayes, 2008). All reported regression coefficients are unstandardized estimates.

### 2.5. Results and Discussions

#### 2.5.1. Statistical Descriptions and Correlations

Table 1 presents the descriptive statistics for both conditions in Experiment 1. Normality tests indicated that all variables deviated from a normal distribution. Spearman’s correlation analyses (see Table 2) showed a positive correlation between cyber-ostracism and bystanders’ helping behavior, with no association with rejection sensitivity.

#### 2.5.2. Manipulation Checks

An independent Mann–Whitney *U*-test showed that participants in the ostracism condition reported significantly higher scores than those in the non-ostracism condition, *Z* = 10.66, *p* < 0.001. This confirms the effectiveness of our cyber-ostracism manipulation in Experiment 1.

#### 2.5.3. The Effect of Cyber-Ostracism on Bystanders’ Helping Behavior

An independent-samples Mann–Whitney *U*-test showed that participants witnessing ostracism reported significantly higher scores compared with those witnessing non-ostracism, *Z* = 7.41, *p* < 0.001. This suggests that bystanders are more inclined to help the target individual who has experienced cyber-ostracism than one who has not.

#### 2.5.4. The Moderating Role of Rejection Sensitivity

The results showed a significant positive prediction of bystanders’ helping behavior by cyber-ostracism, *B* = 0.93, *SE* = 0.11, 95% CI [0.72, 1.14]. Rejection sensitivity did not significantly predict bystanders’ helping behavior, *B* = −0.001, *SE* = 0.01, 95% CI [−0.02, 0.02]. Importantly, the interaction between cyber-ostracism and rejection sensitivity was significant and positive, *B* = 0.04, *SE* = 0.01, 95% CI [0.01, 0.06], indicating that rejection sensitivity moderates the effect of cyber-ostracism on helping behavior. The *R*^2^ of the moderating model was 0.23.

**Figure 1:**
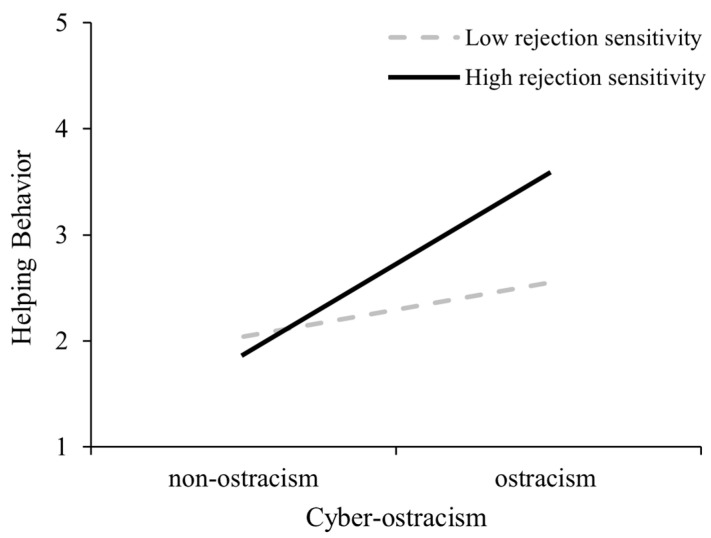
The moderating role of rejection sensitivity in Experiment 1.

Simple slope analyses (see Figure 1) revealed significant differences in the relationship between cyber-ostracism and bystanders’ helping behavior across rejection sensitivity levels. For bystanders with low rejection sensitivity (*M* − 1 *SD*), cyber-ostracism positively predicted their helping behavior, *B* = 0.64, *SE* = 0.15, 95% CI [0.34, 0.94], whereas for those with high rejection sensitivity (*M* + 1 *SD*), this positive relationship was stronger (*B* = 1.22, *SE* = 0.16, 95% CI [0.92, 1.53]).

## 3. Experiment 2

Although Experiment 1 provided initial support for our hypotheses, we conducted a second experiment with two methodological refinements to cross-validate and extend these findings. First, while the text-based scenarios in Experiment 1 established causal relationships, its static presentation may not fully capture the dynamic nature of real-world online interactions. We therefore employed a modified Cyberball paradigm specifically framed as an online ball-passing interaction (for details, see Section 3.2.1). Second, the measurement of “helping behavior” in Experiment 1 was based on self-reported willingness to help, potentially susceptible to social desirability bias. To address this, we measured bystanders’ helping behavior using a resource allocation task, in which sharing tokens with the ostracized target represents a concrete helping behavioral decision (Cialdini et al., 1997; F. Ding & Song, 2017). These refinements enabled us to assess whether the patterns observed in Experiment 1 generalize to contexts with enhanced ecological validity and behavioral concreteness.

### 3.1. Participants and Design

The sample size pre-determination was the same as in Experiment 1. We recruited 258 college students (186 females) aged 18–25 years (*M* = 19.92, *SD* = 1.64) from Yangzhou University via the campus experimental recruitment platform over a 4-week period (18 March to 18 April 2025). All were experimentally naïve (i.e., no prior participation) and randomly assigned to either an ostracism or non-ostracism condition, with 129 participants per group (ostracism: *N*_female_ = 92; *M*_age_ = 19.82, *SD* = 1.49; non-ostracism: *N*_female_ = 94; *M*_age_ = 20.02, *SD* = 1.79).

The participant inclusion criteria replicated those of Experiment 1. This experiment received approval from the Human Research Ethics Committee of Suzhou University of Science and Technology. All participants provided written informed consent and were assured that their participation was voluntary, anonymous, and confidential.

### 3.2. Materials

#### 3.2.1. Manipulation of Cyber-Ostracism

We adapted the traditional Cyberball game (Q. Sun et al., 2025; Williams et al., 2000) to manipulate cyber-ostracism. Unlike the original Cyberball instructions that encouraged participants to vividly imagine real-life interaction scenarios, the present study explicitly directed participants to observe an online ball-passing interaction scenario in the instructions. This modification aimed to precisely align with the conceptualization of cyber-ostracism and emphasize its non-face-to-face nature, rather than evoking real-life interaction imagery. Specifically, participants acting as bystanders learned that three individuals (A, B, and C) engaged in a computer-mediated ball-passing game consisting of 30 passes. In the ostracism condition, participants observed that Player C was initially given two passes and then excluded from further participation. In the non-ostracism condition, participants were told that Player C, along with the other two players, each received 10 passes.

#### 3.2.2. Manipulation Checks of Cyber-Ostracism

The manipulation checks were identical to those used in Experiment 1.

#### 3.2.3. Measurement of Rejection Sensitivity

The measurement procedure replicated that of Experiment 1. Cronbach’s α for this measure was 0.824.

#### 3.2.4. Measurement of Bystanders’ Helping Behavior

Following previous studies (Cialdini et al., 1997; F. Ding & Song, 2017), we used a resource allocation task to measure bystanders’ actual helping behavior. Participants were informed that they would receive 10 tokens, which could be exchanged for monetary compensation. Their task was to decide how many tokens (an integer between 0 and 10) to allocate from their own pool of 10 tokens to Player C, the target individual who was either ostracized or non-ostracized in the observed online interaction. In this context, token sharing constitutes direct compensatory support aimed at alleviating Player C’s interaction deprivation and associated distress. Thus, the number of tokens allocated to Player C served as the quantitative measure of helping behavior, with higher values indicating greater helping behavior.

### 3.3. Procedures

Identical to Experiment 1, participants completed the following in the laboratory in fixed order: (1) measurement of rejection sensitivity, (2) manipulation of cyber-ostracism and its manipulation checks, (3) measurement of helping behavior, and (4) demographic information (gender, age, daily internet usage) collection. The debriefing was identical to that in Experiment 1. The experiment lasted approximately 10 min, with participants receiving an average compensation of 4.20 CNY (≈0.60 USD).

### 3.4. Statistical Analysis

Statistical analysis and processing were identical to those in Experiment 1.

### 3.5. Results and Discussions

#### 3.5.1. Statistical Descriptions and Correlations

Table 3 presents the descriptive statistics for both conditions in Experiment 2. Normality tests (Shapiro–Wilk) indicated that all data deviated from a normal distribution. Spearman’s correlation analyses (see Table 4) showed that both cyber-ostracism and rejection sensitivity demonstrated significant positive correlations with bystanders’ helping behavior. However, cyber-ostracism and rejection sensitivity were not significantly correlated.

#### 3.5.2. Manipulation Checks

An independent-samples Mann–Whitney *U*-test showed that participants witnessing ostracism had significantly higher scores compared with those witnessing non-ostracism, *Z* = 10.74, *p* < 0.001. This suggests that the manipulation of cyber-ostracism in Experiment 2 was effective.

#### 3.5.3. The Effect of Cyber-Ostracism on Bystanders’ Helping Behavior

An independent-samples Mann–Whitney *U*-test showed significantly greater helping behavior in the ostracism condition compared with the non-ostracism condition, *Z* = 3.98, *p* < 0.001. This suggests that bystanders are more inclined to help targets of cyber-ostracism than targets of non-ostracism.

#### 3.5.4. The Moderating Role of Rejection Sensitivity

Results revealed that both cyber-ostracism (*B* = 0.87, SE = 0.18, 95% CI [0.52, 1.23]) and rejection sensitivity (*B* = 0.04, *SE* = 0.01, 95% CI [0.01, 0.06]) significantly positively predicted bystanders’ helping behavior.

**Figure 2:**
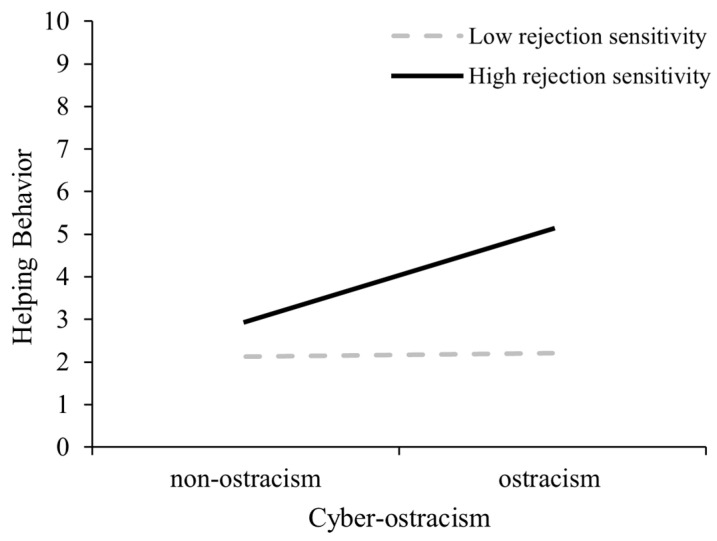
The moderating role of rejection sensitivity in Experiment 2.

Importantly, the interaction between cyber-ostracism and rejection sensitivity was significant and positive, *B* = 0.06, *SE* = 0.02, 95% CI [0.02, 0.10], indicating that rejection sensitivity moderates the effect of cyber-ostracism on helping behavior. The *R*^2^ of the moderating model was 0.25. Simple slope analyses (see Figure 2) revealed that cyber-ostracism positively predicted helping behavior among bystanders with high rejection sensitivity (*B* = 1.45, *SE* = 0.25, 95% CI [0.94, 1.95]), but not among those with low rejection sensitivity (*B* = 0.30, *SE* = 0.25, 95% CI [−0.20, 0.80]).

## 4. Discussion

### 4.1. Interpretation of Findings and Contributions

Using two experiments, this study investigated the effect of cyber-ostracism on bystanders’ helping behavior and its boundary conditions among undergraduates. Results demonstrated that cyber-ostracism significantly increased bystanders’ helping behavior towards cyber-ostracized individuals. Furthermore, rejection sensitivity significantly and positively moderated this effect. These findings not only support our hypotheses but also extend Williams’ (2009) Need–Threat Model by incorporating the bystander perspective, which deepens the understanding of online interpersonal dynamics and offers a scientific foundation for interventions targeting negative online interactions.

#### 4.1.1. Cyber-Ostracism Triggers Bystander Helping

Our study found that individuals witnessing cyber-ostracism exhibited significantly increased helping behavior towards cyber-ostracized individuals, which is consistent with patterns of compensatory behavior observed among bystanders in offline exclusion contexts (Masten et al., 2011; Zou et al., 2022). This suggests that even without physical presence, the interpersonal threat signals inherent in exclusionary events effectively trigger bystanders’ motivation to help victims of cyber-ostracism. This outcome reinforces the core psychological equivalence between online and offline social exclusion (Qin et al., 2025; Williams, 2007). Furthermore, this result aligns with findings from Bernstein et al.’s (2018) online classroom exclusion study, indicating that cyber-ostracism possesses a general property of motivating prosocial responses. This pattern aligns with substituted need–threat mechanisms (Poon et al., 2020; Y. Sun et al., 2023). Specifically, bystanders may internalize the excluded person’s thwarted needs for belonging and control (Petsnik & Vorauer, 2020), subsequently engaging in helping behavior to repair the victim’s impaired self-integrity.

#### 4.1.2. The Moderating Role of Rejection Sensitivity

Importantly, our study revealed that bystanders with high rejection sensitivity displayed stronger helping responses towards cyber-ostracized individuals. This finding aligns with the social-cognitive model of rejection sensitivity (Downey & Feldman, 1996; Levy et al., 2001), indicating that individuals with high rejection sensitivity possess heightened perception of rejection cues. This acuity likely facilitates social projection (Krueger, 2008), mapping their own fearful and painful experiences of rejection onto the victim. This process amplifies empathic perceptions of the victim’s predicament (X. Ding et al., 2020), ultimately driving stronger helping behaviors. Notably, this pattern diverges from findings in second-party interaction contexts (where individuals are direct targets of exclusion). In such contexts, individuals with high rejection sensitivity frequently use social avoidance as a defensive strategy to protect themselves from potential exclusion (Lin et al., 2023; London et al., 2020; Watson & Nesdale, 2012; Zhang et al., 2021), which in turn decreases their likelihood of helping others. However, in the current third-party bystander context, individuals with high rejection sensitivity may empathize more with the excluded person’s helplessness due to their own similar experiences of harm. Assisting cyber-ostracized individuals can help bystanders restore their own sense of belonging and self-esteem while also meeting social-developmental needs through the maintenance of positive interpersonal relationships. Consequently, their motivation to help is significantly enhanced. This finding extends rejection sensitivity research from “excluded person response” to “bystander intervention,” revealing its potential role as a facilitator of prosocial behavior in specific contexts. 

Moreover, we observed that the significant positive effect of rejection sensitivity on bystanders’ helping was present only in Experiment 2, while the positive effect of cyber-ostracism on helping among bystanders with low rejection sensitivity was primarily evident in Experiment 1. These apparent inconsistencies are reconcilable and deepen our understanding of the underlying mechanisms. First, the helping behavior measured in Experiment 1 (i.e., reporting willingness to help) involved minimal cost to participants, as it did not require sacrificing experimental rewards. In contrast, helping in Experiment 2 (i.e., sharing tokens) required sacrificing personal resources, representing a higher cost. This suggests that the helping responses of individuals with low rejection sensitivity are more susceptible to behavioral costs—readily elicited in low-cost contexts (e.g., reporting willingness) but inhibited when costs are higher (e.g., resource sacrifice). This aligns with loss aversion theory (Kahneman & Tversky, 1979), indicating that individuals with low rejection sensitivity prioritize avoiding personal losses in helping decisions. Conversely, the potent social projection mechanism in individuals with high rejection sensitivity appears effective in overcoming behavioral cost thresholds, enabling stronger helping behavior even in high-cost situations. Second, compared with the static scenario description in Experiment 1, the interactive network ball-tossing task in Experiment 2 likely induced stronger emotional arousal, potentially amplifying response differences between individuals with varying rejection sensitivity levels. This highlights the importance of considering the ecological validity and dynamic nature of experimental paradigms when studying the impact of individual differences (e.g., rejection sensitivity) on behavior.

### 4.2. Limitations and Future Directions

While this study yields valuable insights, several limitations warrant mention. First, although we proposed mechanisms (need frustration and social projection) based on theory and prior research, we did not directly measure or experimentally manipulate these constructs. Future research should incorporate mediator assessments (e.g., trait empathy and perceived-need thwarting) or experimental manipulations (e.g., altering bystanders’ need states or projection tendencies) to provide more direct causal evidence. 

Second, the mean manipulation check scores under ostracism conditions fell below the scale midpoint (*M* < 3 on 5-point scales) across both paradigms—the text-based social media interaction (Experiment 1) and the dynamic online ball-passing interaction (Experiment 2). This aligns with evidence showing that observers systematically underestimate targets’ distress (e.g., ostracism) severity (Nordgren et al., 2011), suggesting observer paradigms constrain vicarious emotional engagement to subthreshold levels. Future research on bystander effects should employ more dynamic or immersive methods (e.g., virtual reality) to enhance affective resonance in cyber-ostracism contexts.

Third, while we focused on rejection sensitivity as a key moderator, bystander responses are likely influenced by other factors such as perceived costs, group norms, and situational dynamics. Future studies should integrate these variables to build a more comprehensive model of online bystander intervention. Fourth, our findings are grounded in Chinese undergraduates; their generalizability to non-student populations or diverse cultural/educational contexts remains to be tested. 

Finally, this study examined immediate reactions to a single instance of witnessed cyber-ostracism. In real online environments, individuals may repeatedly encounter exclusion events. Does prolonged or repeated exposure lead to habituation (e.g., numbing), enhancement, or a shift in bystander responses (e.g., withdrawal)? Future research should investigate the temporal dynamics of bystander behavior following recurrent exposure to cyber-ostracism.

## Figures and Tables

**Table 1:** Descriptive statistics for both conditions in Experiment 1.

Cyber-Ostracism Group	Ostracism (*N* = 138)		Non-Ostracism (*N* = 138)		Total (*N* = 276)	
	*M* ± *SD*	Median	*M* ± *SD*	Median	*M* ± *SD*	Median
Rejection sensitivity	56.48 ± 7.00	56.00	57.75 ± 8.56	57.00	57.12 ± 7.84	56.00
Manipulation check scores	2.71 ± 0.75	2.67	1.60 ± 0.62	1.67	2.16 ± 0.88	2.00
Helping behavior	3.08 ± 0.86	3.00	2.17 ± 0.94	2.00	2.62 ± 1.01	2.67
Age	19.74 ± 1.39	19.00	19.70 ± 1.53	19.00	19.72 ± 1.46	19.00
Daily internet usage (hour)	7.91 ± 2.98	8.00	7.30 ± 2.69	8.00	7.61 ± 2.85	8.00

**Table 2:** Spearman’s correlations for variables in Experiment 1.

Variable	1	2	3	4	5	6	7
1. Rejection sensitivity	1						
2. Cyber-ostracism	−0.02	1					
3. Manipulation check scores	0.01	0.63 ***	1				
4. Helping behavior	0.07	0.45 ***	0.55 ***	1			
5. Gender	0.18 **	−0.02	−0.05	−0.004	1		
6. Age	−0.13 *	0.03	0.02	0.06	0.11	1	
7. Daily internet usage (hour)	0.10	0.09	0.03	0.06	−0.06	−0.09	1

Note. cyber-ostracism: non-ostracism = 0, ostracism = 1; Gender: male = 0, female = 1; * *p* < 0.05, ** *p* < 0.01, *** *p* < 0.001.

**Table 3:** Descriptive statistics for both conditions in Experiment 2.

Cyber-Ostracism Group	Ostracism (*N* = 129)		Non-Ostracism (*N* = 129)		Total (*N* = 258)	
	*M* ± *SD*	Median	*M* ± *SD*	Median	*M* ± *SD*	Median
Rejection sensitivity	57.43 ± 9.27	60.00	57.96 ± 9.23	59.00	57.70 ± 9.24	60.00
Manipulation check scores	2.96 ± 0.80	3.00	1.67 ± 0.69	1.67	2.32 ± 0.99	2.00
Helping behavior	3.48 ± 1.66	3.00	2.64 ± 1.54	2.00	3.06 ± 1.65	3.00
Age	19.82 ± 1.49	19.00	20.02 ± 1.79	20.00	19.92 ± 1.64	19.00
Daily internet usage (hour)	7.50 ± 2.86	8.00	7.36 ± 2.92	8.00	7.43 ± 2.89	8.00

**Table 4:** Spearman’s correlations for variables in Experiment 2.

Variable	1	2	3	4	5	6	7
1. Rejection sensitivity	1						
2. Cyber-ostracism	−0.02	1					
3. Manipulation check scores	0.24 ***	0.67 ***	1				
4. Helping behavior	0.36 ***	0.25 ***	0.35 ***	1			
5. Gender	0.08	−0.02	−0.06	−0.06	1		
6. Age	−0.17 **	−0.04	−0.05	−0.09	0.03	1	
7. Daily internet usage (hour)	0.08	0.03	0.01	0.03	−0.03	−0.03	1

Note. cyber-ostracism: non-ostracism = 0, ostracism = 1; Gender: male = 0, female = 1; ** *p* < 0.01, *** *p* < 0.001.

## Data Availability

The raw data are openly available in the Open Science Framework repository at: https://osf.io/hscx5 (accessed on 22 June 2025).
